# “I Felt Like There Was Something Wrong in My Brain”: Growing Up with Trauma – How Young People Conceptualise, Self-Manage and Seek Help for Mental Health Problems

**DOI:** 10.1007/s40653-024-00650-5

**Published:** 2024-09-09

**Authors:** Louise Lynch, Anne Moorhead, Maggie Long, Isobel Hawthorne Steele

**Affiliations:** 1 School of Communication and Media, Faculty of Arts, Humanities and Social Sciences, Ulster University, York Street, Belfast, County Antrim BT15 1ED Northern Ireland; 2 School of Communication and Media, Institute for Nursing and Health Research, Ulster University, York Street, Belfast, County Antrim BT15 1ED Northern Ireland; 3 School of Communication and Media, Centre for Communication and Media Research, Faculty of Arts, Humanities and Social Sciences, York Street, Belfast, County Antrim BT15 1ED Northern Ireland; 4 School of Applied Social and Policy Sciences, Faculty of Arts, Humanities and Social Sciences, York Street, Belfast, County Antrim, BT15 1ED Northern Ireland

**Keywords:** Young people, Mental health experiences, Stigma, Help-seeking, Trauma

## Abstract

**Background:**

Youth mental health is an important global healthcare topic and early interventions that are timely and evidence-based to support young people can increase quality of life and lower deaths by suicide. Research exploring young people’s mental health experiences and how they manage can further understanding into help-seeking processes.

**Objective:**

This study aimed to explore young people’s experiences of living with and managing mental health problems and how this impacts professional help-seeking.

**Methods:**

Eighteen young people were recruited, aged 16–25 years, with experiences of help-seeking to services for mental health problems (*N* = 18). Data were analysed using *Constructivist Grounded Theory* methods (Charmaz, *Constructing grounded theory*, 2014).

**Findings:**

The findings were presented across *three* sub-categories: (1) “*Early experiences”*; (2) “*Conceptualising mental health”* and (3) “*Managing mental health”*. Findings expand understanding on the resource pressures that young people experience whilst managing persistent mental distress emanating from early experiences of trauma, life stressors, and developmental tasks. Findings also report lived experiences of pain, loneliness and stigma, and how individual conceptualisations of mental health are informed. The preference for self-reliance can be rooted in developmental needs or learned behaviours and how this impacts self-management and help seeking is discussed.

**Conclusion:**

Through an enhanced understanding about how young people experience mental distress, developmental pressure points, marginalisation and stigma, mental health providers can prioritise individualised approaches to healthcare that can both respect a young person’s individual conceptualizations and positively leverage self-management strategies, which can contribute positively to young people’s development, quality of life, and healthcare outcomes.

Youth can be described as a time of transition, instability and insecurity due to regular and rapid changes in personal, cognitive, physical, social and sexual development (Best & Ban, 2021; Hochberg & Konner, 2020). The definition of *youth* varies across cultures due to regional economic conditions (UNESCO, 2010; WHO, 2022) but generally describes an individual’s developmental status; that they have begun key physiological and sexual maturation processes but have not yet reached the psychological, cognitive, and economic markers that would classify them as having established ‘adulthood’ (Arnett, 2023; Best & Ban, 2021; Hochberg & Konner, 2020; Mehta et al., 2020). In high income countries *youth* typically includes two primary life-stages, *adolescence* (10 to 19 years) and *emerging adulthood* (20 to 29 years) (Arnett, 2023; Patel et al., 2007; WHO, 2022). An important health issue for this demographic regards mental health problems, which can lead to lower quality of life, disability, educational difficulties, and negative consequences for physical health in adulthood (Bilsen, 2018; Finkelhor et al., 2015; Patel et al., 2007; Pearce et al., 2019; Pompili et al., 2003; WHO, 2022). Additionally, suicide is directly linked with mental health problems and was reported in 2019 as the fourth leading cause of death among 15- to 29-year-olds globally (WHO, 2019, 2024). Suicide is considered preventable, and it often occurs during moments of crisis (WHO, 2024). Accordingly, to reduce this outcome and improve mental health for young people, an important strategy is to provide mental health interventions during childhood and adolescence when mental health conditions are beginning to emerge (Solmi et al., 2022; Kessler et al., 2007).

Research has established that an important contributor to mental health disorders in an individual can be the experience of negative events early in the life span, before 18 years of age (Kessler et al., 2010; McKay et al., 2022). Single traumatic events or ongoing adverse life events can be sources of intensive or frequently occurring stress that can overwhelm ability to cope (WHO, 2020; Zhang et al., 2020) and can result in distress that can lead to mental health problems later. Supporting access to interventions for young people when signs of mental health problems first emerge requires a young person to be able to understand their symptoms and seek help accordingly (Cornally & McCarthy, 2011). In a healthcare context *help-seeking* can be described as both a skill (Rickwood et al., 2005) and a process in which an individual actively seeks help from another to solve a problem (Cornally & McCarthy, 2011). Help-seeking for mental health problems is considered to be an essential coping mechanism (Chan, 2013) and an important approach in dealing with the impact caused by early adverse experiences (WHO, 2020). Young people have been described in research findings as unwilling, avoidant or reluctant to seek help for their mental distress associated with mental health problems (Goodwin et al., 2016; Gulliver et al., 2010), often delaying help-seeking until symptoms of mental distress become severe (Biddle et al., 2007). Reviews of research investigating young people’s mental health help-seeking behaviours have identified multifarious personal, social, cultural, and service factors that can act as barriers or facilitators to mental healthcare (Goodwin et al., 2016; Gulliver et al., 2010; Michelmore & Hindley, 2012; Nam et al., 2010; Radez et al., 2021; Rothì & Leavey, 2006; Rowe et al., 2014). Service factors are reported as pertaining to waiting lists, lack of rural provision, financial barriers and gate-keeping (Cox et al. 2024; Hernan et al., 2010; Leavey et al., 2011; Quinn et al., 2009; Rickwood et al., 2005, 2007; Westberg et al. 2020, 2022). The primary and most reported social barrier involves stigma and cultural or community expressions of mental distress (Goodwin et al., 2016; Gulliver et al., 2010; Michelmore & Hindley, 2012; Nam et al., 2010; Rothì & Leavey, 2006; Rowe et al., 2014), and the role of family and friends are regarded as central in youth help-seeking pathways (Lynch et al. 2023). There are two important concepts of interest for this research, both of which are connected with personal factors affecting help-seeking. The first regards young people’s preference for *self-management*, which can also include topics of self-reliance or autonomy (Biddle et al., 2007; Burlaka et al., 2014; Loureiro et al., 2013; Rowe et al., 2014), and generally refers to an individual’s ability to manage their mental health to produce a satisfactory quality of life (Omisakin & Ncama, 2011). The second concept, *mental health literacy*, describes how an individual recognises problems, describes and manages emotions, as well as their knowledge about treatment and services (Gulliver et al., 2010; Kutcher et al., 2016; Rickwood et al., 2007). Both concepts are central to help-seeking theory with self-management often being both described as a precursor to help-seeking (Chan, 2013) and as implicated in low help-seeking rates, alongside lower mental health literacy (Goodwin et al., 2016; Gulliver et al., 2010).

The global body of literature on the topic of youth mental health help-seeking has provided important insight, but there is a need for further inquiry into young people’s lived experiences of managing mental health problems. Furthermore, additional understanding is required on the impact of childhood contexts on mental health problems, how young people make meaning of personal pain and how these mental health experiences shape their individual conceptualization of mental health and help-seeking processes (Law et al., 2020; Rayner et al., 2018). To do this, it is important to understand how young people learn about mental health and become aware of their distress, and how they move from self-management of mental health problems to wanting external support from a service. Furthering knowledge on this topic can help address gaps in the literature base on how to provide responsive and appropriate healthcare interventions with young people (Lynch et al. 2024), which can contribute towards improved quality of life, reduction of negative life outcomes and suicide prevention (Bramesfeld et al., 2006; O’Neill et al., 2012; O’Neill & O’Connor, 2020; WHO, 2023).

## Aim and Scope of This Study

This study aimed to explore young people’s (aged 16–25 years) experiences of living with and managing mental health problems and how this impacts professional help-seeking, through four objectives. The first objective was to inquire about how young people’s childhood experiences contribute to mental health problems during youth, and the second one was to explore how young people experience and manage their mental health problems. The third objective sought to examine how young people conceptualise mental health and how this influences their approach to mental health problem management. The fourth objective was to explore the relationship between self-management and help-seeking for mental health problems. For the parameters of this research, discussion on “young people” or “youth” can include people aged 10 to 29 years approximately, with adolescence and emerging adulthood being discussed separately when possible. The term “mental health problem” is used throughout and includes the spectrum of personal distress and mental conditions that can negatively affect an individual or cause challenge to well-being (Lynch et al. 2021), and “help-seeking” is used to describe when a young person seeks external support with the aim of lowering their mental distress (Lynch et al. 2024).

## Methods

### Research Design

This qualitative study used methods derived from Kathy Charmaz’s *Constructivist Grounded theory (CGT)* approach (2014), which offered a systematic set of guidelines for facilitating an in-depth inquiry into young people’s mental health experiences to obtain rich data. This study’s aim and objectives were best achieved using this design as it supported inquiry into the personal, social, structural, historical, and cultural meanings of an individual’s reality, essential factors that determine their beliefs, actions, and attitudes, all of which can shape mental health experiences (Charmaz, 2014; Golafshani, 2003; MacKenzie & Knipe, 2006).

### Study Participants

This research study took place in the Northwest of Ireland with young people aged 16–25 years of age with experiences of help-seeking for their mental health problems. Young people who had attempted, exited early or completed a help-seeking episode were welcome to take part. Participant characteristics are described in Table 1.

**Table 1:** Youth participant demographics (*N* = 18)

Age	Gender	Location	Ethnic background	Education or employment status
16	M	Urban	Irish	Student
16	F	Rural	Irish	Student
18	M	Gaeltacht	Irish	Student
18	Tm	Urban	European	Student
19	M	Urban	Polish	Student
19	F	Urban	Irish	Student
19	F	Urban	Irish English	Employed
21	F	Urban	Irish	Student
22	F	Urban	Irish	Employed
22	F	Urban	Irish	Unemployed
23	F	Urban	Irish	Unemployed
23	M	Urban	Black African	Employed
23	M	Urban	Black African	Employed
24	M	Rural	Irish	Employed
25	M	Rural	Irish	Employed
25	F	Urban	Irish	Employed
25	M	Gaeltacht	Irish	Employed
25	M	Urban	Irish	Employed

*Gaeltacht, is the term used to refer to those areas of Ireland where the Irish language (Gaeilge) is the primary spoken language of the majority of the community

The research findings were part of a larger study that collected qualitative data on youth mental health help-seeking with young people (*N* = 18) and mental health practitioners (*N* = 6). As this article focuses on young people’s experiences of living with and managing mental health, only data from youth participants (aged 16–25 years) were reported (*N* = 18).

### Participant Recruitment

**Fig. 1:**
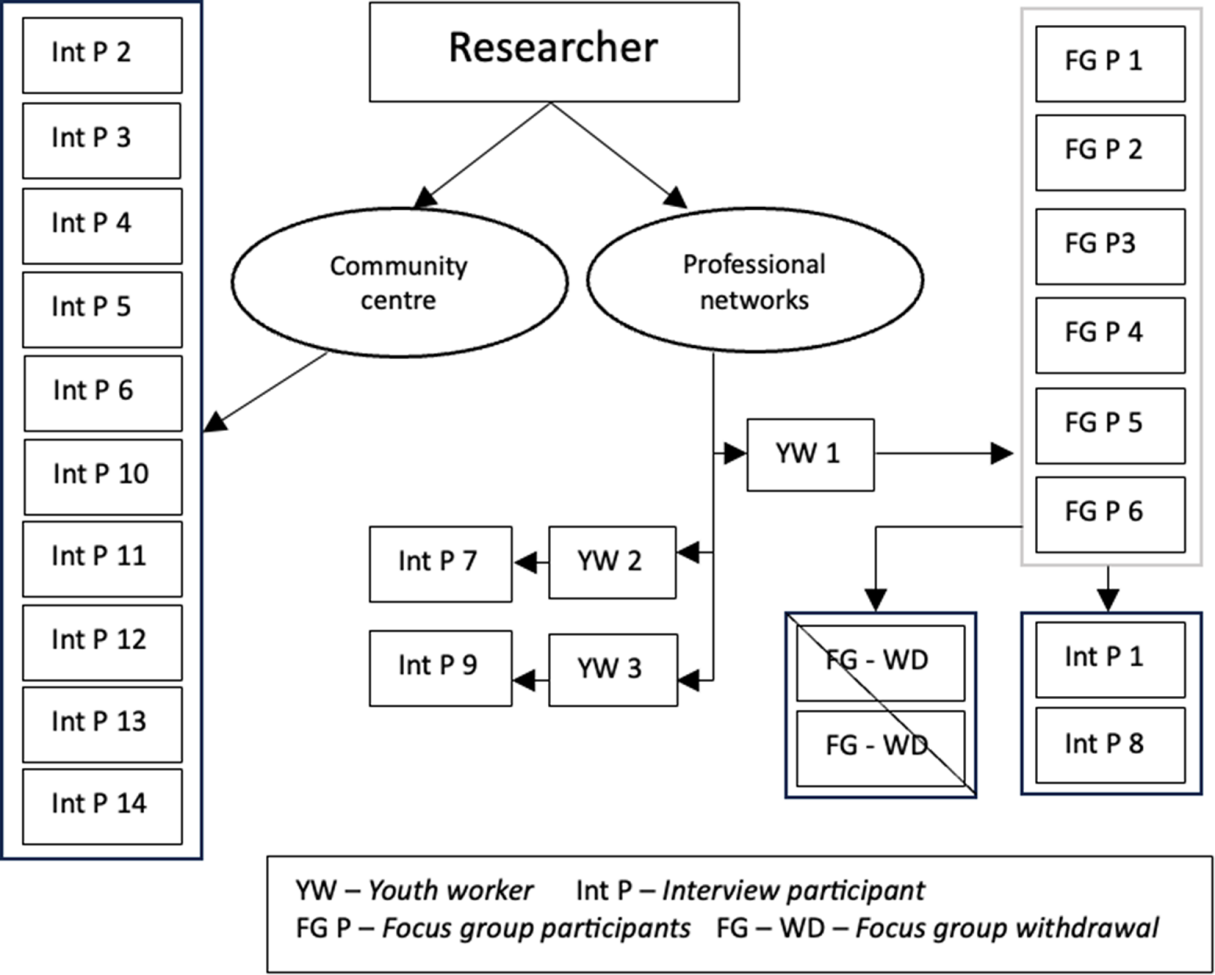
Sampling pathways

A combination of *purposive sampling* and *snowball sampling* strategies were employed to support the recruitment of young people with a diversity of perspectives and experiences between the ages of 16–25 years (Clark et al., 2021). Snowball sampling is a relational approach where participants are recruited through existing networks, which can support rapport building and encourage trust (Barbour & Barbour, 2003; Ghaljaie et al., 2017). Participants were recruited in two ways, firstly the researcher advertised the research in a community service drop-in space and engaged in conversations with interested individuals. Secondly, information sheets were distributed through existing staff networks and were passed on to interested individuals (Fig. 1).

Two young people withdrew before the focus group commenced and two focus groups participants also volunteered to do an interview, which meant that two participants took part in both a focus group and an individual interview. In total, eighteen participants (*N* = 18) took part in one focus group (*N* = 6) and interviews (*N* = 14). This included young people aged 16–19 years (*N* = 7) years and 20–25 years (*N* = 11).

#### Participant Selection

As the focus of this research was to explore experiences of living with mental health problems, self-management, and professional help-seeking, participants were required to be able to reflect on the personal journey of experiencing and self-managing mental distress that became significant enough for them to initiate help-seeking with a professional mental health service, and included those who made one contact, exited early or completed a help-seeking episode. Interested individuals were provided with a participant information sheet and decisions to participate were made in partnership after a conversation with the researcher, to ensure participants thought that they were suitable for the research. Those wishing to participate in this research self-reported that they met inclusion criteria (Table 2) and then self-selected to participate in an interview or focus group.

**Table 2:** Selection criteria

Inclusion	Exclusion
Aged between 16 and 25 years	Have not sought help for a mental health problem with any service
Have sought help with a formal service or semi-formal service with a minimum of one contact	Have been referred directly by a practitioner/service employee to the study
Have sought help with a formal or semi-formal service within the previous four years	Have an intellectual disability
Is not currently in crisis or in the early stages of receiving mental health support	Not able to provide consent

*Formal mental health services* were defined as providers and professionals with a specified role in delivery of mental health care such as counsellors, psychologists, psychiatrists and mental health nurses and similarly, *semi-formal mental health services* included providers and professionals who encounter or provide support with those who need mental health care as part of other duties, typically school guidance counsellors and youth workers (Lynch et al. 2024). Young people in crisis or the early stages of receiving mental health support were excluded for ethical reasons as participation could cause further distress due to the intensity of crisis experiences and/or the impact of potentially speaking about unprocessed and distressing emotions connected to mental health experiences.

#### Ethical Considerations

Ethical Approval was obtained from the Ulster University Research Ethics Committee (UUREC No: 180010) and data storage adhered to the General Data Protection Regulation (2018) and Data Protection Act (2018). This research followed The British Psychological Society’s (BPS) *Ethical Principles for Conducting Research with Human Participants* (2014) ensuring voluntary participation and informed written and verbal consent was obtained. Given the topic under exploration and the potential for distress, written and verbal briefing and debriefing were provided which included local mental health services. The researcher was trained in child safeguarding and had access to a support structure for safeguarding concerns although no safeguarding issues were disclosed during this research. Additionally, while a distress protocol was developed, it was not used as no participant became distressed during data collection. Participant demographics (Table 2) were deliberately disconnected from the pseudonyms used throughout the findings as an additional anonymity check.

### Data Collection

Data collection took place in a community-based service meeting room between May 2018 and December 2019. The focus group and interviews were audio recorded and the duration was between 20 min and 1 h 10 min. Participants were welcomed, offered refreshments and consent was discussed and agreed before beginning. Interview participants were guaranteed confidentiality, however, due to the nature of a group discussion, the limits of confidentiality in a focus group context were explained to participants before obtaining verbal consent and commencing data collection. All participants were appropriately debriefed. The decision to stop was informed when the sampling technique was not providing any new properties, also known as saturation (Charmaz, 2014).

### Interview and Focus Group Guides

Semi-structured interview and focus group guides were devised to explore young people’s direct experiences of living with and managing mental health problems. Along with the aim and objectives, concepts from the literature review, such as ‘help-seeking’, ‘self-management’ and ‘mental health literacy’, informed the development of the guides. The terms presented at the beginning of this article were discussed with participants before beginning data collection so that both could engage in a discussion with defined parameters. Additionally, Bronfenbrenner’s Ecological Systems model (1995) supported the researcher to consider how questions could be framed to best explore how young people self-manage and help-seek for mental health problems within and across systems, and the influence of wider social, cultural and service factors. Finally, *theoretical sampling* was used, which is an approach that involves adding questions on themes that are identified during data collection and posing them to subsequent participants (Charmaz, 2014; Lambert & Loiselle, 2008). Using both the CGT technique of intensive interviewing (Charmaz, 2014) and the flexible yet focused guides facilitated deeper exploration on the topic to uncover new meanings for both the researcher and the participants (Bryman, 2012; Galletta, 2013) and sample questions, including examples of probing questions from participant transcripts are available in Table 3.

**Table 3:** Question guide: sample questions

	Prepared guide sample questions	Sample probing questions or added questions
Introductory questions	• What is your understanding of the term mental health? • What does mental health mean to you? • Where did you learn about mental health?	• How did your understanding of mental health change as you got older? • If you weren’t taught about mental health, how did you know you needed help?
Core questions	• When did you first begin to experience difficulty with your mental health? • How long did it take before you realized you had a problem? • What is it like to be a young person with a mental health problem? • What do you find helpful/unhelpful when coping with distress? • When did you decide to ask others for help? • Were you hesitant about telling others or seeking help for your mental health? • Was there anything that caused you to wait longer/act quicker? • Have you heard of other people’s experiences of asking for help and have they influenced your decisions?	• What exactly made you realise that how you were feeling wasn’t ‘normal’? • Can you tell me more about what it was like to be seven years old and have those feelings? • How did your family respond? • Are there some examples that you feel comfortable talking about or just naming? • What helped you cope during that time? • What was happening for you during that time when you thought ‘I just can’t manage on my own anymore’? • What did that distress feel like? • Was your distress ongoing or did it culminate in that week? • What held you back? Why didn’t you go seek help the next day? • Can you tell me a little bit more about the stigma and embarrassment and shame you just described around having a mental health problem? • How did you unlearn that stigma? • You sought help at 21, had you previously tried when you were a teenager or child to tell someone you weren’t feeling okay? • How did not having access to support during your childhood impact you at 25? • Would you know other people your age who have experienced similar things?
Closing questions	• Based on your experience would you go to a professional mental health service again? • Is there anything you would like to say that you haven’t had the opportunity to say yet?	• How has this been for you talking about all of this? • Are you feeling alright?

#### Methodological Triangulation

Vandermause (2007) emphasises the need for innovative methods and combinations of qualitative methods or *methodological triangulation* to address the complexity of healthcare phenomena. Moreover, this approach can be used in qualitative research to support trustworthiness and quality (Creswell & Miller, 2000; Golafshani, 2003), to elucidate multiple aspects of the phenomena explored (Tobin & Begley, 2004) and for a richer analysis (Morse et al., 2002). Additionally, *methodological triangulation* enables participants to self-select to either a focus group or an interview, supporting individual preference and comfort levels (Lynch et al., 2018). Interviews can offer focus and privacy, however, some individuals prefer to discuss healthcare topics in a group setting and thus focus groups are an interactive and developmentally effective method for facilitating discussions about mental health (Gibson, 2007). Focus group data can also provide interactive and narrative data around how experiences are constructed and created in group settings (Lambert & Loiselle, 2008; Litosseliti, 2003). Finally, in data analysis, this methodological combination was used for data completeness, and provided insight into the similarities and differences of the group (social) and the individual (subjective) realities (Lambert & Loiselle, 2007, 2008; Morse et al., 2002).

### Data Analysis

Data analysis is a process that is embedded in all stages of the CGT approach (Charmaz, 2014). Data were transcribed in sequential order and were organised and coded systematically using Nvivo 12 by the lead author, with transcripts and coding reviewed by the second and third authors. Data that contained rich descriptions of young people’s early life or childhood experiences, mental distress or trauma experiences, conceptualisations of mental health, self-management strategies and how these influenced help-seeking actions, were coded for. The first stage of coding used a combination of *word-by-word*, *line-by-line*, and *incident with incident* techniques which supported the researcher to look for subtle meanings and insights, alongside the extensive sorting and organising of codes (Charmaz, 2014). The next stage of *focused coding* organised codes into categories and sub-categories that formed the overall theoretical concept of “young people’s mental health experiences”. The researcher utilised many other CGT techniques, such as *familiarisation with data*, *memo writing*, and the *constant comparative method*, which all supported an in-depth analysis of interviews and focus groups (Charmaz, 2014). Each data set (interviews and focus groups) was initially analysed separately and then cross analysed revealing predominantly complementary data (Lambert & Loiselle, 2008).

#### Integrity in Research

The research addressed highly sensitive topics and was conducted using a trauma-informed approach in partnership with participants from initial contact to debriefing, to avoid unintentional objectification, retraumatisation or discomfort and facilitate relevant, meaningful and authentic conversations around mental health experiences. Furthermore, rapport development, positive regard, care, and active listening were prioritised to each participant (Prior, 2018) and a carefully prepared interview guide combined with great sensitivity and a well-managed interactional space created a safe environment that respectfully gathered rich data (Charmaz, 2014; Lambert & Loiselle, 2008; Mackay, 2012; Prior, 2018; Richards, 2020). Participants reported positive experiences of participation and follow ups or *member checking* (Creswell & Miller, 2000) was utilised as a trustworthiness check, where the researcher shared the results with some participants (Creswell & Miller, 2000). One participant reported that they valued reviewing research findings and how reading about others’ experiences validated their own mental health experiences. Using the guidelines from Galletta (2013) and Charmaz (2014), the first author engaged in *reflective practice* throughout the research. Other important trustworthiness checks included *peer debriefing* (Creswell & Miller, 2000) with co-authors, the provision of a clear and thorough description of the design (Golafshani, 2003; Lambert & Loiselle, 2008) and analysis grounded in the provision of rich descriptions of data in the findings section (Creswell & Miller, 2000).

## Findings

**Fig. 2:**
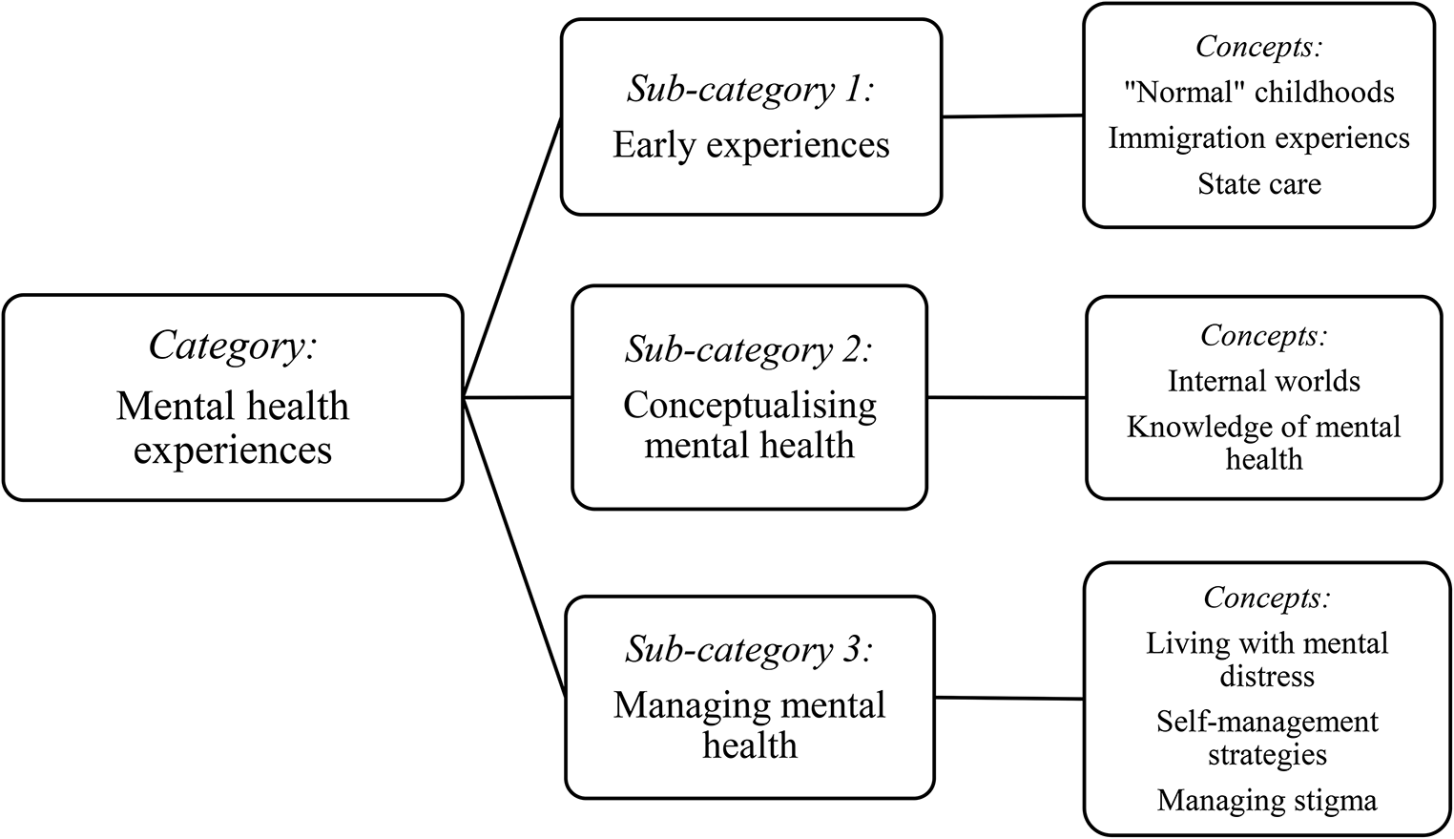
Young people’s mental health experiences

This research explored eighteen young people’s (aged 16–25 years) experiences of living with and managing mental health problems. In total, eighteen young people participated in interviews (*N* = 14) and a focus group (*N* = 6) (Table 2). Participants were asked about their earliest experiences with mental health, and they volunteered what information they were comfortable with disclosing, which included descriptions or references to mental distress beginning during childhood. It was notable that more female than male participants were able to recall or possibly were more comfortable sharing childhood experiences. Some young people spoke of specific traumatic events or described ongoing stressors during childhood and adolescence that overwhelmed their coping (Zhang et al., 2020), with all participants reporting that these experiences contributed greatly to their mental health problems in adolescence and emerging adulthood. This early life context of trauma experiences was an important finding in this study and participant reports included: *caring responsibilities for parents or siblings*,* parental bereavement*,* parents with mental health problems or addiction*,* exposure to drug or alcohol use in the community*,* deprived communities*,* community/peer/family suicide*,* transgenerational trauma*,* parental abandonment*,* domestic violence*,* family separation*,* divorce*,* asylum seeking and immigration experiences*,* threat due to identifying as LGBTI+*,* racism*,* loneliness*,* bullying*,* neglect*,* poverty*,* educational disruptions*,* physical abuse*,* emotional abuse*,* sexual abuse*,* homelessness*, and *state care*. The findings present rich data on young people’s lived realities of growing up with trauma experiences and living with and managing mental health problems, which are presented across three sub-categories: *early experiences*,* conceptualising mental health*, and *managing mental health* (Fig. 2).

### Sub-Category 1: Early Experiences

#### “Normal” Childhoods

All young people discussed events and circumstances during childhood which caused intense or ongoing distress: “I started having panic attacks when I was about five or six” (Laura). Some young people spoke about the difficulty in trying to understand and process significant life changes such as family separation, divorce and moving house: “… being that young and not understanding, it seems far worse than it is, because I don’t have the pieces to put together” (Claire). Other participants described childhood abuse, living in rural and deprived environments impacted by problems with alcohol and drugs, or living in unsafe communities with predatory adults: “but nobody did anything” (Laura). A few young people discussed experiences of parental abandonment, caring for parents, and/or neglect due to caregiver mental health or addiction:

In my mom’s case she did not want to admit that she had depression or whatever it was, she’d tell us she was getting help but she wasn’t and I think that’s the reason for her losing it [psychosis description] (Gerard).

Laura reported experiencing parental bereavement and the subsequent caring roles she acquired: “I went there [school] to get a break because I would go home and then I would get to work”. Also discussed was the long-lasting impact of unmet needs for love and positive attention and poor-quality family or caregiver attachments: “You want to feel loved by somebody, it’s a natural thing and you’d get put down for it, you’d be told ‘stop looking for attention” (Cathy). Mental health problems in adolescence and emerging adulthood were described by participants as an inevitable continuation of the persistent and unresolved distress they experienced as children:

I’ve always experienced problems, from about seven years, I remember just wanting to hurt myself and you never know what it is, you just think you’re a crazy little kid …but then that carried on through and got worse as I got older (Cathy).

At seven I saw a lot of crazy stuff… and then when I was maybe 14, I just remember feeling so alone (Thomas).

#### Immigration Experiences

Five young people in this study reported experiencing immigration to Ireland during childhood or early adolescence. Thomas discussed being a child in a family that sought asylum and described feeling overwhelmed and powerless due to this experience, as well as managing other types of distress such as parental mental health problems, cultural differences and alienation due to racism: “… their [adults] biases are very real and it’s not like I wanted to come to your country … it wasn’t my decision, it was out of our hands”. Thomas also described how traumatic experiences before this transition: “I’ve seen [pauses] … I was a kid and I saw a man being killed in front of me…” combined with asylum seeking experiences contributed to mental health problems during adolescence. Andrew also discussed his experience of immigration to Ireland from an African country as a result of a private fostering arrangement between his parents and another African family established in Ireland. Andrew described feeling disconnected, isolated and lonely during childhood and adolescence due to changes in fostering agreements, instability in foster homes and having to rely on his own resources:

it’s just different from everyone else who has got either a mum or a dad and families but for me it’s just [pauses] it’s just you and your [siblings] … with no parents … there’s so many stuff you go through as a young person (Andrew).

James and Rachel both reported immigrating to Ireland during their teenage years to live with extended family. James discussed feeling unsafe, unwell, and unaccepted in his home country due to extreme right-wing political views:

…my health was deteriorating, because I live in [home country], it’s not very friendly environment for LGBT people, I was still in the closet… I was very tired of pretending and came to Ireland hoping that I could find some acceptance (James).

Rachel, who had experienced caregiver addiction and abandonment during childhood, immigrated in adolescence with the hope of connection, identity and family care: “I was so excited but six months later I was in a new school in a new country, and I didn’t know anybody, am [pauses] and I became very suicidal”. Young people discussed how immigration could provide some immediate physical safety or access to a psychologically safer environment but how additional distress from displacement or relocation compounded existing distress.

#### State Care

Two young people in this study reported substantial exposure to adversity during childhood that resulted in them spending time in care of the Irish state during their childhood and adolescence: “…any kid in care is going to have issues already. You didn’t end up in care for the fun of it” (Áine). Gerard described growing up with social work involvement as he had caring responsibilities for a parent. Upon turning 18 years of age Gerard reported losing this professional support and shortly after experiencing family crisis, suicidality and homelessness: “It was a very difficult week”. For those who entered the care system, the safeguarding experience that separated them from their family was described as a deeply traumatic transition:

Social workers have the power to take away children, they don’t look at the emotional side of it … after the safeguarding is done, their job is done – they just leave you to wonder about all of these depressing feelings (Rachel).

Áine described her deteriorating mental health whilst living in a residential setting with limited confidentiality, privacy, or opportunities for developing trusting relationships. She discussed social workers as prioritising professionals’ needs in the mental health services needs over hers, and reported feeling pathologized, and the impact of constant staff turnover:

…even when it’s not a child protection issue it still goes back, I could have told them ‘I think the sky is green’ and they’re repeating what I said, so you don’t trust them in that sense, and then you kind of give up on them because … the person always changes (Áine).

Participants with care experiences identified that ‘being in care’ had caused further adversity due to unstable living placements and instability in mental healthcare, which hindered the development of support networks and undermined educational attainment. When the time came to leave care at 18 years, this transition was described as retraumatising:

I think my biggest one [barrier] was actually being in care, because I moved so much, by the time they were getting the referrals sent I was being moved… and it’s no wonder I ended up in crisis so many times (Áine).

Overall, participants childhood experiences, including their adversity, was experienced as normal to them and became central in shaping their understanding of their distress and in what way they conceptualised mental health.

### Sub-Category 2: Conceptualising Mental Health

#### Internal Worlds

All participants described mental health as a normal but complex part of human experience and distress was reported as an expected aspect of existence: “it’s a fact of life you know?” (Liam). Young people struggled with defining mental health: “the mind is a terribly confusing thing” (Liam), but agreed that it comprised of emotions, feelings, intrapsychic processes and the abstract or intangible aspects of the self, which were all connected to physical health. Erin succinctly stated: “it’s the part you can’t see” and described it as a “lens” which affected her cognition, memory, and perception. Some participants conceptualised fluidity or duality: “… you can have bad and good at the same time” (Andrew) and many described division and conflict, between the self as experiencing the distress, and the brain as inflicting the distress independently:

It’s like – I can win this battle myself, but that wasn’t the reality … it took a few years for me to finally give in to the illness and just say I need something else [therapy] because this [self-management] is not working (Joseph).

All participants discussed their understanding of mental health problems as being culturally pervasive: “It is like the common cold, everyone in Ireland is touched by it in some way” (Joseph), a perception that could increase comfort, connection and reduce feelings of personal defectiveness:

Everyone I know is a bit fucked up [laughs] and I include myself in that statement, we’re all a bit weird, we fit in because we don’t fit in and it’s nice (Gerard).

In general, conceptualisations of mental health were directly impacted by how young people acquired knowledge on the topic and what they observed from others in their family and social networks.

#### Knowledge of Mental Health

Most participants described learning about mental health independently or through friends and family: “…we were never taught about mental health or anything like that” (Áine). Due to the normalisation of mental distress in childhood, some participants reported that they were not able to identify distress as a problem that could be helped:

Someone said to me once, ‘I think you’re just having a panic attack’ and I was like – what? [laughs] Are you being serious? I thought it was a normal part of my life! (Laura).

Difficult or abusive home environments as well as family mental health problems could also be normalised during childhood: “when I became a teenager, I realised that there was an awful lot going on in my family and I just couldn’t deal with it on my own” (Erin).

As mental distress increased, some young people reported experiencing a noticeable behaviour changes, which as Rachel described, was often first flagged by someone else: “I was told that I needed help”. Others were essential in supporting the development of self-awareness of distress and its origins, a process that typically began during mid- to late adolescence but was reported as more commonly developing during emerging adulthood after positive help-seeking experiences: “… I only developed that about two years ago” (Andrew). However, connecting with others about traumatic experiences could cause some young people to unhelpfully compare circumstances, which could trigger feelings of shame: “… it just makes yourself feel worse and guilty and shame for feeling the way you do… it might just end up causing you to go into that hole even deeper” (Erin). A few participants described learning during adolescence about mental health as resulting from a personal defect or chemical problem, one that could potentially be “fixed”, however, helpful interactions with mental health professionals supported this conceptualisation to evolve:

…I wanted help to stop it, I needed somebody to analyse it and to be like ‘okay this is what’s wrong here and we’re going to put you on this and we’re going to get you help … [pauses] they can’t stop the flashbacks…but I understand that now (Laura).

The combination of early experiences and knowledge of mental health was found to be central for informing how mental distress could be managed.

### Sub-Category 3: Managing Mental Health Problems

#### Living with Mental Distress

The daily reality of living with mental distress for many young people was characterised as living between oscillating periods of acute distress and relief or respite: “I would have a few months where I would be bad and a few months where I would be good” (Laura). Participants who reported substantial life stressors also reported more intense cycles or living with constant mental distress without respite which Rachel described as: “just surviving and that’s it”. Increasing tension or pressure led to peaks or crisis points where “you kind of explode” (Rachel). Cycles generally intensified and some participants reported using self-injury, substances or attempting suicide during peaks, as a means to cope with mental distress.

All participants described how their sleep, eating, self-care, interest, motivation, relationships, and planning for the future were affected: “just insomnia, depression, everything so you’re like, this is really shit” (Cathy). Consequently, managing mental distress could be isolating and negatively affect attendance at school and/or employment. Laura described reducing hours at work due to flashbacks that were triggered during therapy: “It ruined work for me going to counselling”. Gerard described feelings of hopelessness and misery while trying to manage mental health problems and homelessness on his own: “I didn’t have anything; everything was just shit and I was on my own and everything was awful”. Many participants also spoke about managing alone because of the limited capacity of family or friends to provide support and Erin spoke about trying to manage family mental health problems on top of her own: “I was just thrown into it – there’s depression, suicide in our family”.

Developmental transition points particularly around puberty transition (11 years approximately) and the legal transition (18 years approximately) were described as expected challenging stages that were more difficult to navigate with mental health problems: “The teenage years are complicated and add in mental health, that’s adding a whole universe of complications” (Áine). Additionally, many participants described the extra pressures on mental health from technology and social media: “We’re not fully equipped to understand how to cope with that and it just leads us to internalise stuff or have issues within ourselves” (Erin), as well as growing up during the 2008 Global Financial Crisis, and trying to manage anxieties against the backdrop of global issues. Liam described his generation as profoundly affected by these issues, which could undermine motivation to address mental stress:

Constant existential dread … its constant low-level worry for me that the way things are going with climate change, the rise of horrible global inequality and the international fascist movement, things are getting pretty bleak … I have tried to get my own internal house in order, but as much as that helps and as much good as you do, [pauses] I don’t personally see things getting better (Liam).

Many young people described how coping with adversity in childhood caused earlier development in some areas such as problem solving or stress management but that it could also delay some aspects of personal or social development: “…almost like I was an adult before I was a teenager” (Claire). Laura spoke about her caring role for her family members and missing out on socialising or time for play, and how self-managing persistent mental distress over years had contributed to the normalisation of her circumstances and her emotional pain: “I didn’t realise that it was something that I could get help for” (Laura). Self-management strategies were idiosyncratic to participants but contained common approaches and were often connected to coping styles developed in childhood.

#### Self-Management Strategies

All participants described the management of their distress as their responsibility and that self-management was their initial preference: “I like to deal with things by myself” (Laura). Furthermore, self-management was perceived as an important developmental goal, crucial for self-esteem and self-efficacy: “I just want to feel better on my own [without medication] and I was never able to vocalise that” (Rachel). Common and helpful short-term self-management strategies included withdrawal to avoid having to manage other’s reactions: “I need to be alone and I can’t let anybody see it” (Laura) or alternatively, suppressing or masking pain to ensure continued participation in daily life, especially in attending school: “I am the master of faking stuff like you wouldn’t believe” (Claire). Participants also described behaving aggressively to release tension: “I was a **bitch** in school for a while and if the teacher started on me by God, I would finish it” (Claire), and self-harm to help manage distress: “… I’d beat the shit out of myself, and I couldn’t sleep at night unless I did” (Laura).

Liam discussed cultural lesons on how to manage distress: “go say your confession and have a pint, job done, [laughs]”, and how his frequent use of drugs and alcohol to cope with intensifying distress eventually led to embarrassment and increased shame: “I would have the occasional drunken breakdown with friends… that happened more and more when I was going through some really bad stuff”. Some participants discussed using denying, minimising, and distraction through humour, watching TV, video games, or waiting for distress to pass: “Just play football and forget about it? [Laughs]… but it’s still there” (Thomas). These strategies were often advised or learned from family or friends who often promoted avoidance, stoicism and concealment: “and that has been passed down, because they didn’t talk about it – we don’t talk about it now” (Claire). Families could also promote escapism, distraction or industriousness: “my cousin would call me every day and tell me ‘go do that course’ but I wasn’t interested and taking part would make me more miserable” (Gerard). Participants also described observing how families and communities managed the impact of suicide and the understanding that was communicated in the absence of explicit conversation that mental health problems could result in suicide: “here [North West of Ireland] it’s a silent epidemic and people don’t talk about it” (Joseph).

Young people described using different but reliable types of self-management strategies, both reflexive and learned, to manage mental health problems. Many participants spoke about using exercise and finding enjoyment through making plans. Others praised the role of art, music, creativity, or writing: “I found writing and making stories the way that I express my emotions” (Laura). For some young people, changing environment could offer important economic and social opportunities and many participants discussed the importance of trusting relationships and connecting with others’ experiences: “I feel when I meet someone who has the same thing as me, or is relatable… you have **real ** conversations, they’re nearly as good as counselling and they’re free!” (Joseph). Regular offloading was specifically described by participants as helping increase capacity to return to their expected social and sexual development: “I was sort of like okay, but I wasn’t at the same time, but I was going through that stage where I thought – I look amazing, all the boys want to kiss me!” (Claire). Additionally, Cathy described how knowing help was available supported self-management in the short-term: “I’m feeling shit now, but it’s okay, I can go and talk to someone tomorrow!”. Positive experiences of professional help had supported some participants with improved self-management, self-care strategies and earlier intervention in future distress: “If you feel yourself starting to get down it’s best to kind of deal with that now and acknowledge that that’s happening” (Liam). However, managing persistent mental distress alone over long periods had a personal impact: “It led to a very painful road” (Joseph) and most participants discussed making decisions by late adolescence to seek help due to the increasing ineffectiveness of self-management strategies.

#### Managing Stigma

All participants described different types of stigma as important factors influencing self-management strategies and avoidance of help-seeking. Learning about what behaviours were stigmatised occurred when help-seeking to family and friends in late childhood and early adolescence: “the Irish family being… ah sure there’s nothing wrong with you… you’ll grow out of it” (Cathy). Additionally, mental distress could be stereotyped or invalidated as ‘typical’ or gendered teenage behaviour: “… if you’re female it’s seen that, well you’re just a teenager, you’re being a drama queen, your emotions are just all over the place, it’s puberty” (Áine). Cathy reported avoiding professional help-seeking due to previous negative experiences with family stigma: “…because of your experiences in childhood where it’s ‘don’t talk about it’, coming into your teenage years, coming out to an adult is a lot harder because you don’t want to be shamed again by an adult…”. Laura specifically discussed avoiding help-seeking because of stigmatised generational labelling: “the ‘snowflake generation’ and the ‘millennials’ … you don’t want to show anyone else that you’re hurting, especially if they make fun of you for being a cry-baby…”.

The historical legacy of asylums on Irish culture was reported as still impacting societal mental health stigma: “…like ages ago you would have been locked up if you had anxiety… and obviously we have gotten better but it’s not where it should be” (Claire). Áine also discussed the impact of growing up in a culture with legacies of institutional abuse and transgenerational trauma. Irish cultural norms were described as modelling the concealment of problems, which were also evident at a structural and governmental level through the avoidance of action or accountability of harm of historical abuses:

… we like to be seen as being a friendly and accepting country and if that means brushing everything under a carpet, we will, and we have for centuries, and we’re very good at it. We’re not good at admitting faults and actually rectifying them – ‘okay we’re sorry we did this like that 50 years ago’ – [moves hands] under a carpet. Gone. Instead of going ‘right I’m sorry we done this, let’s do something about it’ (Áine).

Laura also spoke about how the Catholic Church, and their authority across personal and public life, helped cement a culture of concealment, shame and self-stigma:

… constantly under a cloak of shame, they don’t talk about it and then nobody wants them to talk about it … it is very bad for mental health and people don’t realise how closely linked the church is with a lot of that stuff (Laura).

Some participants internalised stigma and labelled themselves as “freak” or “crazy”, and usually expressed this safely with humour: “I was half functioning two years ago and I was a ball of neuroses held together by substance abuse! [laughs]” (Liam). Others expressed feelings of defectiveness: “I felt like there was something wrong in my brain” (Laura) and how even thoughts of help-seeking could trigger or intensify distress and cause damage to an already impacted self-image: “I don’t know how to explain this, like a **shame**, you feel like you’re being ungrateful, you feel embarrassed … the stigma is like you’re weak” (Thomas). Laura explained how anxiety and depression are more accepted mental health problems, often attributed to thinking styles or personality traits and are less stigmatised than other types of distress: “…people think you’re really fucking crazy if you have got PTSD, because people think if you have anxiety, you’re just a worrier”. Managing persistent mental distress throughout childhood and adolescence impacted many participants’ identity development:

This is the person I have become … I don’t know what I’m like when I’m not like this. I’ve grown up with it, so it is already embedded in me… I don’t know if I want to get better, I don’t know if people will like me when I’m myself (Claire).

Andrew described how when supported appropriately, especially in later adolescence, earlier trauma experiences and subsequent distress could be reframed positively and contribute to positive self-image in emerging adulthood: “…and I think when you actually go through some stuff … and you get **past **it … you go woah, you’re actually a lot stronger than you think” (Andrew). Andrew also described successfully reframing help-seeking as an autonomous and a decisive self-management strategy: “There is a lot of ways that you can help yourself, number one is definitely talking to someone… talking to people would be a way that I would deal with it”. Stigma was an important mediating factor in how participants made decisions to continue with self-management or seek help, and young people often waited until self-management was ineffective and distress was high before fully engaging with help-seeking processes and reframing conceptualisations positively.

## Discussion

This research explored young people’s (aged 16–25 years) experiences of managing mental health problems during youth. All young people in this study discussed mental distress as beginning during childhood (Kessler et al., 2007; Solmi et al. 2022) and reported subsequent difficulties with effectively identifying, labelling and communicating needs or feelings with their caregivers. Descriptions of early help-seeking attempts during childhood by seeking comfort and positive attention or by expressing sad feelings were discussed as frequently dismissed by caregivers. Additionally, some caregivers were described as missing distress entirely, especially when feelings were internalised (Loureiro et al., 2013). In particular, young people reported how experiences of traumatic events were frequently met with silence (Downes et al., 2013), dismissal or an inadequate discussion, which resulted in them being unable to understand or process these experiences and the associated distress. It was often when young people described externalising behaviours during adolescence or emerging adulthood, for example, aggression, self-harm or substance use, that others noticed distress and initiated conversations about mental health, thus beginning the process of learning how to communicate, label, self-manage and seek help for their mental health problems more effectively.

### Developing with Distress

**Fig. 3:**
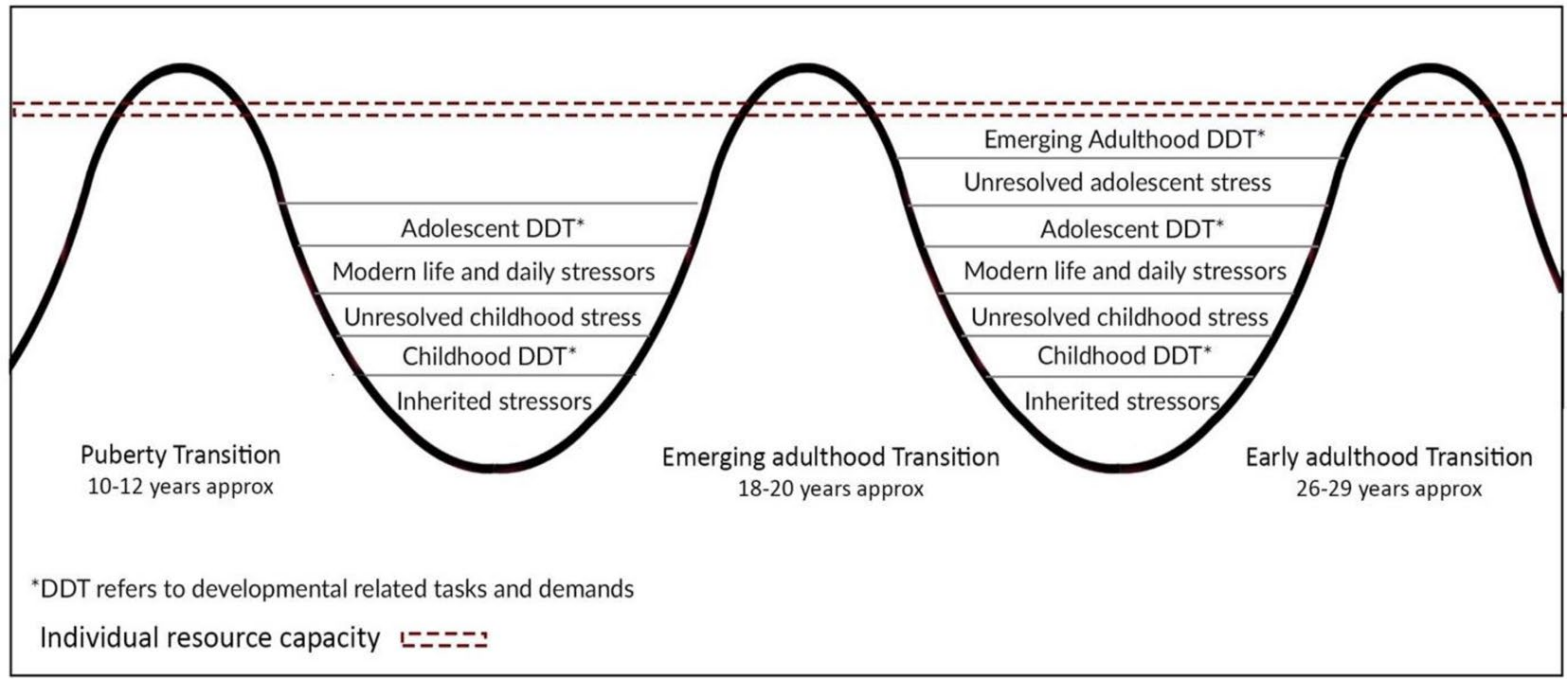
Developmental pressure points and individual resource capacity in young people

Participants discussed managing persistent mental distress during childhood using their personal resources and individual coping strategies, typically until early adolescence, when expected developmental challenges put pressure on their already stretched individual resource capacity. Figure 3 was developed from the findings to illustrate how managing mental health problems connected with unresolved or unprocessed experiences from early adversity can use the cognitive, social, and emotional resources needed for other normative developmental or expected life stressors, especially around key developmental transitions, which can create pressurised distress points.

*Daily life stressors*, broadly refers to the ongoing and somewhat expected issues related to social, educational or economic challenges, and *modern life* stressors acknowledges the extra pressures of living in a technological and globalised society, for example in the managing of social media or the existential impact of climate change. *Inherited stressors* describe the wider family or community issues that can contribute to a young person’s mental distress, such as transgenerational trauma (Downes et al., 2013) and toxic cultural or societal legacies, for example living in a community that has experienced extensive institutional abuse. Figure 3 presents these stressors equally for illustrative purposes but the actual impact or weight of each of these pressures can be specific to an individual’s context, the stressor load they carry, the degree of childhood adversity experienced, and their family and community’s resilience (Hadfield & Ungar, 2018), all of which affects their individual capacity. During transition phases, a young person can reach their individual resource capacity limit and experience intense distress or breach this capacity and enter *crisis*. Managing mental health problems could mean developmental demands were suppressed or paused, as individual resources were diverted to the more immediate demands of managing emotional pain and problem-solving to reduce distress or the circumstances contributing to it. While young people can consider help-seeking at any point it was found to occur more frequently when they were reaching their individual resource capacity limit as self-management was less effective and mental distress was reported as increasingly interfering or overwhelming. This experience of stressor overload could trigger thoughts around seeking external support as a management strategy and this finding contributes understanding to previous studies that report young people as generally highly distressed when first help-seeking for their mental health (Biddle et al., 2007; Burlaka et al., 2014). Furthermore, Pillemer et al., (2007) found that negative self-esteem memories in adulthood are often related to memories of interpersonal conflict that have been carried through from childhood and adolescence, and this research found similar findings, that young people moved through their development with distress acquired from earlier stages. These findings reaffirm the need to consider the connectedness of mental health across the life-stages of the individual (Rayner et al., 2018) and the impact of persistent mental distress in early life on development, self-image, identity, and self-esteem into later life.

### Living with and Managing Distress

Mental distress was experienced by young people as painful (Sachs-Ericsson et al., 2017), disruptive, and as affecting global functioning and quality of life (Meade, 2016). Participants described managing difficult feelings daily, which included anxiety, depression, obsessive or intrusive thoughts, flashbacks, sadness, loneliness, shame, anger, bodily disgust, and self-loathing. Young people described experiencing mental distress as a constant state or as fluctuating waves of tensions that were managed until they passed or were released via an outlet. Recognising this pattern, young people waited for distress to pass or expected it to disappear (McSherry et al., 2015) and indeed distress for many did temporarily pass however, it also tended to return and to exacerbate over time due to unresolved mental health problems and mounting developmental or new life-stage challenges.

Young people described using both the reflexive and learned strategies that they acquired during childhood and adolescence, to self-manage distressing emotional states such as rage, horror, despair, shame, panic, and chaos. This included constructive and destructive approaches to coping that included socialising, exercise, creative activities, aggression, violence, self-harm, control, distraction, alcohol, and drugs (Burlaka et al., 2014; Jorm et al., 2008; Rickwood et al., 2007). When young people’s mental distress was connected with interpersonal conflict or social rejection, *withdrawal* could prevent further distress from the source of their pain – other people, and isolation provided young people with respite from the resources required when having to mask pain publicly. *Concealment* of distress supported engagement with social activities and protection from stigma, which could also prevent damage to public image from pejorative terms such as “snowflakes” or “soft” or “weak” (Alyeksyeyeva, 2017; Chan, 2013; Wrathall, 2017). However, in the longer term, these strategies could result in further distress such as feeling disconnected, different, defective and lonely. Loneliness is considered prevalent in adolescence with some studies suggesting it as normative due to many reasons such as social restructuring and genetics (Wong et al., 2018). In this research, loneliness in young people was found to directly stem from managing difficult feelings and other age-related problems on their own. Some young people can fear social rejection for their perceived defectiveness, which can also lead to self-stigma and further concealment of problems. Loneliness and self-stigma were described as relieved with positive help-seeking experiences, when young people connected with others who listened and provided empathy or who related well, especially if they experienced similar problems (Law et al., 2020). Moreover, young people can also use humour and self-labelling, such as ‘crazy’, as a way of releasing tension and reducing self-stigma (Corrigan et al. 2014; Cross, 2013).

During crisis points young people described managing suicidal thoughts, panic, rage, powerlessness, hopelessness and intense emotional pain similar to physical pain, and thus help-seeking was considered a strategy for immediate relief to make the pain stop. However, as key developmental tasks during youth involve identity, autonomy, and role experimentation (Arnett, 2023), thoughts of help-seeking could also trigger additional distress, reinforcing feelings of failure, personal defectiveness, or compromised autonomy. Consequently, delaying help-seeking was reported as a protective strategy to reduce or avoid further distress. The reframing of help-seeking positively occurred later in youth and typically required both the *capacity*, from natural maturation processes, and the *opportunity*, from a supportive other, to disentangle understandings of mental distress as arising from a personal defect rather than from something originating in their environment, namely negative dynamics, interpersonal conflict, or early adversity. Young people described how individual resource capacity was most often restored through opportunities to offload and process difficult feelings with a supportive other. In adolescence, this subsequent increased capacity was not always used for learning new coping strategies or skills but for returning to developmental tasks including making friends, dating, forming identity, planning for the future, or educational and employment related learning (Arnett, 2023; Neilson et al., 2014). Later in emerging adulthood, young people wanted to learn skills or tools to improve self-management strategies as a means for achieving the perceived developmental goals of an independent adulthood, which can include finding full-time employment, further identity development, formalising romantic partnerships and experimenting with roles for established adulthood (Arnett, 2020; Mehta et al., 2023).

Adolescent distress within Western industrialised nations is often normalised and expected, with stereotypes stemming from early ideas of “storm and stress” (Hall, 1904). Findings in this research indicate that mental distress in adolescence can be othered by adults and characterised as sudden, biological, phasal, illegitimate or unremarkable, and this impacts how they live with and manage their mental health problems. Indeed, there are mixed messages across society regarding the normality of mental distress during youth which can enable adults to respond dismissively, especially when distress is not externalised (Loureiro et al., 2013). Young people discussed how national campaigns promoting help-seeking such as “it’s okay not to be okay” could positively de-stigmatise mental health problems but some participants also thought that it also could reinforce minimisation or cause invalidation by promoting distress as normative, as some young people can interpret the slogan in a literal manner. Another perspective on normalisation in mental health is provided by Davies (2018) who advocates that difficult life experiences, which emanate from individual-environmental challenges, are not labelled ‘abnormal’ and the findings of this research indicate that the context of young people’s lives is experienced as ‘normal’ to them. Accordingly, it is important that mental health messaging is communicated carefully and with empathy; young people’s mental distress can be acknowledged respectfully without pathologizing, stereotyping or stigmatising pain, and that ‘normal’ does not mean that pain must be tolerated privately or managed alone.

The impact of legal and economic dependency experienced during adolescence was another important finding that influenced how young people managed their mental health problems. Much of adolescent life occurs in controlled settings (Pearow & Pollack, 2009) where traumatic events and interpersonal problems can occur, such as in the home or in school, and the inability to remove oneself from these environments can cause feelings of imprisonment, oppression, and powerlessness. Youth is a type of legal marginalisation that can be grown out of in most industrialised countries at 18 years of age, but as this legal transition is not aligned with developmental science (Best & Ban, 2021; McGorry et al., 2019), some young people experiencing substantial adversity can be thrust into new forms of marginalisation.

### Compounded Marginalisation in Youth

Marginalisation on top of trauma and persistent mental distress was found to profoundly affect a young person’s personal resource capacity, resulting in further delays to personal and social development. Young people in this study who experienced significant adversity found themselves in emerging adulthood having missed opportunities for important development, which further intensified mental health distress, and discussed the grief and overwhelm they felt when trying to address and learn life skills alone. Life after 18 years of age without family or community support for young people who experienced homelessness, immigration or state care was found to include further adversity, instability, further victimisation, retraumatisation and experiences of living from crisis to crisis.

Young people who experienced state care are at great risk for mental health problems (Fargas-Malet & McSherry, 2018), and findings from this research showed that abuse or neglect from caregivers and the subsequent separation from them were reported as separate traumatic experiences. This separation, and subsequent relocation, caused or triggered feelings of fear, loneliness, rejection, abandonment, and powerlessness, feelings that were retriggered each time they were moved by services, and this highlights the critical need to support young people with this transition (Watson et al., 2020). In addition, placement outside of the birth family environment was typically in a different geographical location from the young person’s community, school, services, and social networks, causing additional distress associated with this displacement. Young people described how being discharged from state care at the legal transition age of 18 years (Damian et al., 2018; McGorry et al., 2019) contributed to instability in living arrangements, inconsistent mental health care and inadequate aftercare, which were found to be common experiences during and after state care that caused further psychological harm and trauma. The outcomes of these experiences included retraumatisation, incomplete education and fragmented social networks, which participants reported as contributing to multiple suicide attempts, in-patient experiences, drug use and/or experiences of homelessness (Collins & Barker, 2009; Narendorf, 2017). These experiences during adolescence and emerging adulthood could entrench feelings of failure, hopelessness and defectiveness and significantly interfere with development, education and employment, of which young people in these circumstances greatly needed for economic independence and stability.

Some young people in this research described immigrating to increase physical safety, improve economic conditions and/or psychological wellbeing. Young people shared experiences of coming to Ireland as part of a family seeking asylum, often having experienced adversity and trauma (Gatt et al., 2020), or during childhood under a ‘kinship foster care’ system (Ejorh, 2012; Palacios & Jiménez, 2009), which can include private arrangements where a child is legally fostered for the purposes of economic and educational opportunities by friends or other family relatives who are already established in Ireland. In these circumstances, findings suggest that young people can experience deep loneliness and mental distress associated with family separation, relocation and poor attachments with foster family, impacts that can be obscured or minimised by the prioritised economic and educational opportunities. In addition, young people who self-identified as Black African in this study discussed the harmful impact of racism on their mental health, especially from adults in institutions, such as teachers and Gardaí (police), and how this undermined trust in wider structures provided in Irish society, including mental health services (Fanning, 2018).

Ireland has a long history of migration to the United Kingdom, America, and Australia, and research has found that mental distress is common among second-generation Irish children (Das-Munchi et al., 2014). This research also found that young people who are second generation Irish can immigrate back to Ireland in the hope of improved wellbeing and to connect with wider family. In addition, immigration within the European Union (EU) is common due to free movement legislation (European Union, 2022), and this study found that young people who are threatened by the increase in anti-LGBTI+ laws in other EU countries can view Ireland as a safe place to immigrate to (Evans, 2014). While declaring LGBTI+ asylum is a practice globally (Jordan & Morrissey, 2013), the findings in this study indicated that this type of asylum seeking can remain hidden across countries with free movement, as can any associated mental health problems. For young people with immigration experiences, the challenges of the geographical and cultural transitions meant that establishing stability was prioritised, resulting in the temporary suppression of mental distress that could re-emerge later for processing when they felt safer. Alternatively, when a young person could not find the type of safety they required (physical or psychological) this circumstance could elevate distress to extreme levels and was reported as a contributing factor in suicide attempts. It might not be evident to practitioners on first meeting with a young person the degree of adversity that is being carried and young people themselves might not always be able to identify or label the multifarious life stressors that have contributed to their mental health problems. Moreover, assessment questionnaires might not always account for or capture the range of experiences that can cause some young people trauma, for example, young people with caring responsibilities, hidden LGBTI+ asylum seeking across Europe or young people in international kinship foster systems, and is a reminder that when working with young people the importance of rapport, time, trust and an individualised approach (Lynch et al. 2024).

### Conceptualising Mental Health

Mental health literacy can be viewed as a life skill (Rickwood et al., 2005) and while a useful term in Western healthcare research, this concept can have limitations due to the differing conceptualisations of mental health that exist globally, locally and personally (Cosgrove et al., 2019). Furthermore, the term *literacy* can imply the existence of a curriculum to be learned and thus individual competency, which could unintentionally locate responsibility with the person for their distress, and judgement for their subsequent problem-solving choices, actions or in-actions. In addition, this term can obscure how experiences inform attitudes, beliefs and actions, and the socio-environmental context where children learn about mental health. Rayner et al., (2018) and Law et al., (2020) have emphasised the need to understand how young people conceptualise the nature and meaning of mental health for themselves, and this research found that using this approach supported the development of understanding on how young people self-manage and seek help for mental health problems.

**Fig. 4:**
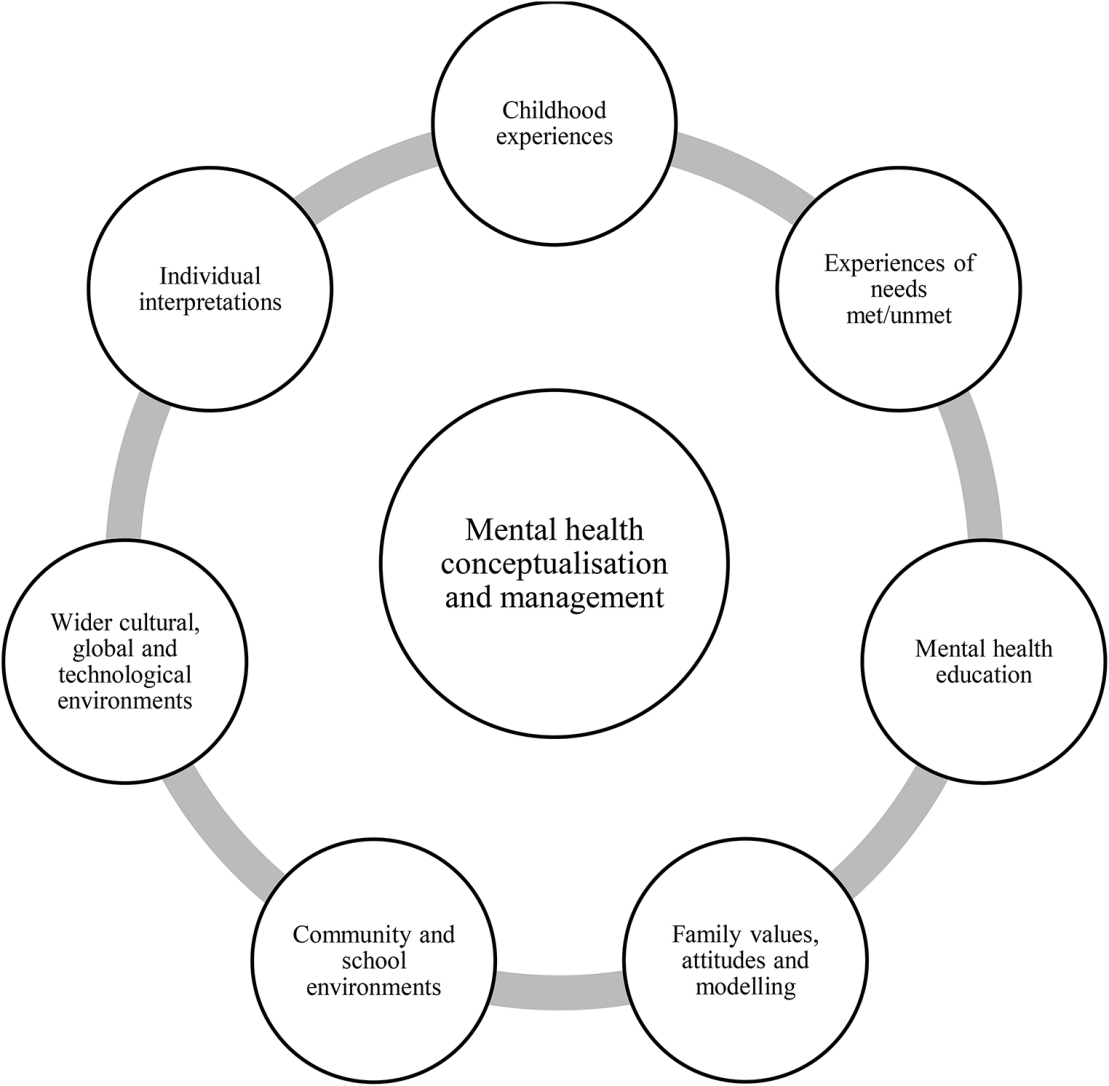
Factors influencing individual mental health conceptualisation

Young people in this research were questioned about their personal conceptualisation of mental health and where they learned about it. There were often long pauses during interviews, with young people expressing challenge, confusion, and duality when trying to define a concept or articulate their perspectives, often reverting to Western physical or clinical health-based paradigms, which had limited explanatory power to encapsulate the entirety of their experience. Mental health was described as a profound and central part of the self, involving identity, with an important mediating role in the overall quality of life. The environment and relationships with the self and others were also central to mental health, and many participants described how their mental health was affected by on-going and complex interactions between personal, experiential, interpersonal and external factors, such as service provision, and across different environments and systems (Bronfenbrenner, 1995). These personalised conceptualisations directly impacted young people’s understandings, memories, ideas, attitudes, and feelings of distress, which informed their problem-solving approaches and associated decisions on help-seeking. This research also found that while young people’s conceptualisations of mental health varied, they shared similar overarching concepts and stigmas. This can be explained by their unique psychosocial and environmental makeup whilst living within common systems and structures (Bronfenbrenner, 1995; Cosgrove et al., 2019; Davies, 2018). Figure 4 was devised from this research to illustrate the broad learning environment from birth that creates a young person’s mental health conceptualisation and can include young people’s childhood experiences, how their needs are met, formal education, family values, community, and wider national and global cultural lessons, and of course their own individual feelings, experiences and analysis.

Young people in this study reported learning coping skills from caregiver modelling, which included predominantly concealment, alcohol, substance use, prayer and stoicism (Moore et al., 2013). These approaches are possibly connected to legitimate fears from a previous time when families lost members exhibiting mental distress, amongst other issues, to asylums or institutions, and thus concealment of distress was a protective strategy to avoid institutionalization, stigma and/or community exclusion (Goodwin et al., 2016; Inglis, 2015; Masuda et al., 2009; Murray et al., 2006). This research also found that young people managed unresolved distress from the previous generations, for example, when living with a parent with mental problems, and young people described how stoicism facilitated concealment (Fargas-Malet & Dillenburger, 2015; Johnston et al., 2013; McLoone-Richards, 2012) meaning that all distress, including the traumatic, remained silent (Long, 2021). Young people reported a ‘knowing’ that their parents and grandparents had experienced trauma, through observations of what was expressed and unexpressed. Empty spaces in conversations were interpreted as containing taboo experiences and this was reported as one method for the transgenerational transmission of mental health stigma in families (Pompili et al., 2003).

This research found that young people have to manage others’ conceptualisations of mental health when help-seeking for mental health problems, including their families, their community and culture, often navigating between different values systems and frameworks of taboo to meet their own needs. Common sources of resilience and support have changed with the rapid social, industrial, and technological changes of modern life (Gatt et al., 2020) and findings show that technology blankets young people’s lives, connecting them with global culture and issues, which are often experienced as the local. This aspect of globalised living requires cultural tools and individual skills to navigate, manage, and integrate into daily living to prevent information overload and technology-related stress. Moreover, the clustering of the discussed factors – history of asylums, legacies of institutional abuse, religious control over private spheres, concealment, inheriting unresolved distress, and suicide – have intersected with modern life and technology, meaning that young people, who are digital natives (Burn et al., 2010), need to have different conceptualisations and tools for coping with mental health problems than those of older generations. Additionally, a young person’s conceptualisation of mental health can be different to those of professionals working in formal services, schools, youth services or other professions that can encounter and support young people with mental distress. Why an individual uses certain coping strategies and how they can be supported to incorporate new information, skills and strategies requires time, rapport and an individualised approach to support the development of their conceptualisation of mental health and approaches to coping (Lynch et al. 2024).

### Self-Management and Help-Seeking

**Fig. 5:**
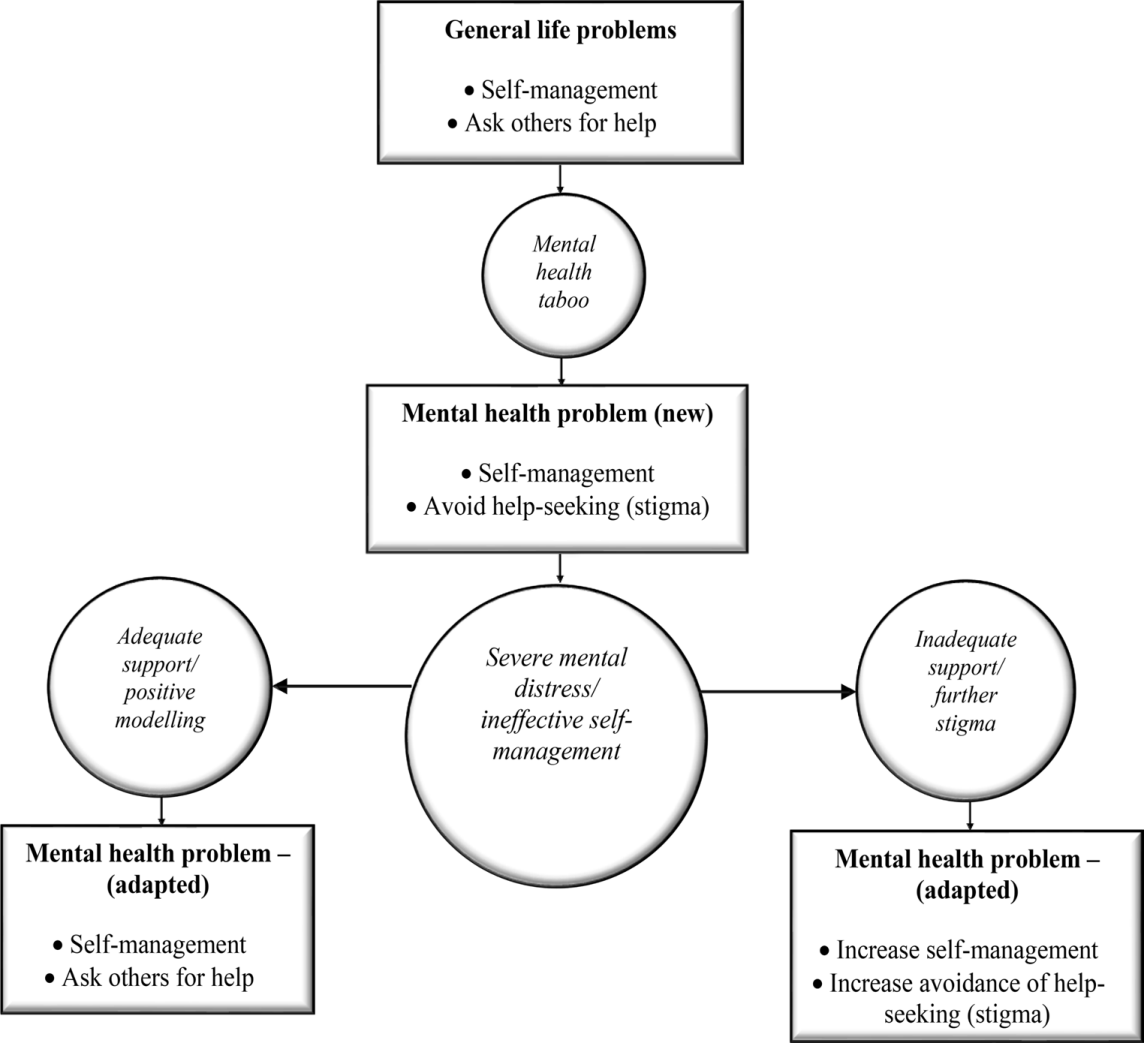
Schema assimilation and accommodation in mental health

As discussed, young people in this study described learning about mental health self-management during childhood from observing others, through the process of modelling (Bandura, 1978). Analysis suggests that when young people (as children) sought comfort or support for emotional distress from others and received a negative response (dismissal, minimisation, punishment or humiliation), they learned that their distress was not valued, that expression was taboo, and that help-seeking could incur stigma, criticism or rejection. Consequently, they learned to accommodate and internalize distress, and *exclude* help-seeking behaviour (and the associated stigma) as a means of problem solving for emotional needs, engaging instead with self-management strategies that were acceptable and that supported self-reliance. This reportedly helped avoid further distress and social rejection (Freedenthal & Stiffman, 2007; Wilson et al., 2005), and participants discussed only considering help-seeking out of absolute necessity (Hickie & McGorry, 2007; Jorm et al., 2006). Figure 5 was developed during this research to illustrate how help-seeking for mental health problems can become excluded from general help-seeking *schemas* in childhood (Piaget, 1952), and how new schemas around distress management can be created that can initially include managing mental health problems alone but can evolve to accommodate help-seeking behaviour as part of a mental health problem management strategy.

Adapting a mental health problem schema to include help-seeking involved re-examining personal, family and community values about coping, and seeking out others who could model and reframe help-seeking positively. Participants described this process as challenging, and generally not possible when they sought emergency mental health care, emphasising the vulnerability of help-seeking during crises, and the re traumatising impact of services who provide inadequate support or who respond with negative or stigmatising responses.

Help-seeking schemas were found not to be deterministic or linear, but dynamic and responsive, with opportunities for amendment in positive and negative directions at every interpersonal interaction, especially during disclosures of distress. Some young people reported descriptions of early positive experiences of help-seeking with services that produced less internalisation of stigma and supported the development of schemas that normalised help-seeking. Additionally, through a supportive rapport, young people developed increased mental health awareness and developed more effective self-management strategies (Omisakin & Ncama, 2011; Rickwood et al., 2007). Alternatively, inadequate or no access to service provision was found to further reinforce previous negative experiences, support hyper self-reliance and entrench coping strategies such as self-harm or substance use. This meant that self-management was often the only available pathway for coping, and this supported the revision of the *mental health problem* schema to accommodate ideas such as, ‘nobody will help me’ or ‘I can’t be helped’, which was found to contribute to hopelessness and the development of suicidal pathways.

Previous research has described “self-reliance” or “need for autonomy” as a *barrier* to help-seeking but this research found that young people’s needs for autonomy were linked to the normative developmental drive of individuation (Arnett, 2023). This drive is connected to the desire to achieve economic independence, one of the markers of established adulthood and is likely rooted in the wider cultural ideals of self-reliance and independence in Western culture (Arnett, 2023; Bramesfield, 2006; Mehta et al., 2020). Practicing self-management through demonstrating competency and avoiding dependency became increasingly important to young people in preparation for adulthood and in providing self-esteem (Arnett, 2023; Wilson & Deane, 2012). This research proposes a distinction between the developmental need for autonomy, an *aspirational state* to achieve and self-reliance as a *learned behaviour*, developed through modelling and from managing distress alone. When others had proved unhelpful or dismissive, beliefs regarding self-reliance were reinforced and self-management became an important and reliable coping mechanism to which thoughts of help-seeking could undermine and upset internal schemas, causing further distress. This research found that autonomy in general could be leveraged to support young people to reframe help-seeking positively without destabilizing their identity (Bilican, 2013; Del Mauro & Williams, 2013; Eisenberg et al., 2011; Masuda & Boone, 2011; Prior, 2012; Yap et al., 2013) and the reframing of help-seeking as a self-management strategy, that the decision to access somebody who has the resources to help manage a problem, can be an act of personal power, agency and autonomy.

### Limitations

This research included young people from diverse backgrounds but was not able to recruit young people from important ethnic communities in Ireland, such as the Travelling and Roma and this limits the findings (Fanning, 2012). In addition, this research took place in Ireland which is a high-income country with Western cultural influences, transferability could be limited to the region or cultures with similar contexts. Finally, this research obtained perspectives from young people who engaged and self-reported experiences and thus the experiences and help-seeking processes of those who for many reasons do not come forward or those who have died by suicide were not heard and could not be included.

### Implications and Recommendations for Further Research, Practice, and Policy

This research inquired about young people’s childhood experiences and how they contributed to mental health problems (objective 1) and their experiences of living with and managing their mental health problems (objective 2), contributing important findings to the wider literature on lived experiences of pain, loneliness and stigma. Additionally, findings expand understanding on the resource pressures that young people experience whilst trying to manage persistent mental distress, expected life stressors, and developmental tasks, which can cause disruption or even pauses to development.

This research also examined how young people conceptualise mental health (objective 3) and found multifarious factors that influence individual conceptualizations and how this can subsequently impact self-management and help-seeking decisions. This research develops our understanding of the concept of mental health literacy, and how our understandings as researchers and practitioners can support or restrict how we interpret youth mental health help-seeking behaviours and subsequently treat young people.

Finally, this research explored the relationship between self-management and help-seeking for mental health problems (objective 4) and reports that avoidance of help-seeking can be connected to both a developmental need for autonomy (*aspirational)* and/or from managing mental distress alone during childhood (*learned behaviour).* Moreover, *mental health problem-solving* schemas were found to be informed by modelling, and the effect of family, community or societal stigma and this could mean young people do not always internalise help-seeking as an acceptable strategy until otherwise shown how to. These findings elucidate further how delayed help-seeking can be understood as a protective strategy and not just a barrier, to prevent further distress and damage to self-image (Chan, 2013), and additionally, that help-seeking as a concept, can also be positively reframed as an autonomous decision and a decisive action, with positive messaging and modelling.

Recommendations for policymakers and practitioners include providing young people with consistent care across the life-stage of youth (Solmi et al., 2022) and to provide interventions that support young people with unprocessed distress, specifically approaches that first restore individual resource capacity (offloading, active listening) before engaging in building capacity (problem solving, coping strategies). This is especially important for young people managing distress and marginalisation associated with homelessness, caring responsibilities, immigration or state care. Adolescents can prioritise using their restored resources to address developmental needs, and thus might not fully engage with capacity building activities until later in emerging adulthood when improved self-management strategies are needed to support economic independence goals. Furthermore, practitioners can consider in what way young people are self-managing (aspirational autonomy/learned behaviours) and appropriately guide and support autonomy when devising treatment plans and goals (Lynch et al. 2024). Additionally, it is also important in treatment planning to consider that the range of experiences that can be sources of intensive or frequently occurring stress that overwhelm a young person’s ability to cope are not always adequately captured in assessment due to limitations in tools or the young person’s normalisation of circumstances and distress.

Future research can consider exploring young people’s experiences of early life stress as well as and negative life and marginalisation experiences that can cause trauma, for example, hidden LGBTI+ asylum seeking across Europe or young people in kinship foster systems internationally. Future research can also continue to explore young people’s contexts and help-seeking processes with an aim to develop youth specific help-seeking frameworks that captures the complexity of help-seeking pathways during youth. Finally, research can consider how to improve youth mental health messaging with particular attention to those who have mental health problems but who have not yet sought help, and to consider co-design methodologies to ensure research is conducted in partnership with young people.

## Conclusion

This research explored young people’s (aged 16–25 years) experiences of living with and managing mental health problems (*N* = 18) using a constructivist grounded theory approach (Charmaz, 2014). Young people can experience trauma during childhood and suffer persistent mental distress, which without support or intervention, can be carried into adolescence and emerging adulthood, negatively affecting development. This research contributed insight into what it is like to live with mental distress and has examined young people’s self-management strategies and how they learn about and conceptualise mental health. Young people often only develop the ability to identify and label distress effectively during adolescence and emerging adulthood, and self-reported high levels of distress when seeking help. Additionally, analysis critically examined concepts of mental health literacy and self-reliance with regard to young people’s wider development. Through an enhanced understanding about how young people experience mental distress, developmental pressure points, marginalisation and stigma, mental health providers can prioritise individualised approaches to healthcare that can both respect a young person’s individual conceptualizations and positively leverage self-management strategies, which can contribute positively to young people’s development, quality of life and healthcare outcomes.
